# There Is No Longer a Role for Systematic Biopsies in Prostate Cancer Diagnosis

**DOI:** 10.1016/j.euros.2022.01.006

**Published:** 2022-02-10

**Authors:** Veeru Kasivisvanathan, Mark Emberton, Caroline M. Moore

**Affiliations:** Division of Surgery and Interventional Science, University College London, London, UK; Department of Urology, University College London Hospitals NHS Foundation Trust, London, UK

The ideal prostate cancer diagnostic test should identify clinically significant prostate cancer that would benefit from treatment, avoid diagnosis of insignificant cancer that does not benefit from treatment, and have a low side-effect profile.

Systematic transrectal ultrasound-guided (TRUS) biopsy, the mainstay of prostate cancer diagnosis for the past 30 yr, is associated with errors leading to clinically significant cancers being missed and overdetection of clinically insignificant cancer that might not benefit from treatment but can lead to overtreatment-related harms. This may contribute to why randomised treatment trials such as PROTECT and PIVOT, which used systematic TRUS biopsy as the method for cancer diagnosis, have shown limited benefit of radical treatment in improving prostate cancer-specific mortality [1].

Systematic TRUS biopsy alone should no longer be carried out as the primary diagnostic test in men with suspected prostate cancer. The PRECISION study demonstrated that a pathway involving magnetic resonance imaging (MRI) first followed by MRI-targeted biopsy (MRI-TB) in men with an MRI-visible lesion leads to more significant cancer being identified, fewer insignificant cancers being diagnosed, and nearly a third of men avoiding biopsy altogether [2]. Similar benefits, particularly in reducing the detection of insignificant cancer, have been demonstrated widely in other studies such as PRECISE and MRI-FIRST [3].

An additional question is whether one should add systematic biopsies to MRI-targeted biopsies or perform MRI-TB alone. A well-conducted prospective clinical trial showed that an additional 5% of Gleason 3 + 4 cancer cases would be detected on addition of systematic biopsies, although only 1% of these were Gleason 4 + 3 or worse [3]. There are a number of major methodological limitations for the within-patient study designs in the literature that will tend to overestimate the relative performance of systematic biopsies. Test-review bias is likely as the operator performing the systematic biopsies is typically aware of the location of the MRI lesion, so that the systematic biopsies are somewhat “targeted”. Furthermore, most of these studies limit MRI-TB to three or fewer biopsies. There is growing evidence that increasing the number of cores per target results in an enhanced yield of clinically significant cancer [4]. There are further data indicating that the learning curve for MRI-TB is more than 100 procedures [5], yet hardly any of the studies in the literature report on experience level [6].

Even if some low-volume Gleason 3 + 4 disease is missed by an MRI-TB–alone strategy and is “MRI-invisible”, there are data showing that nonvisible Gleason 3 + 4 disease has a different biology to MRI-visible, clinically significant detected cancer. MRI-visible prostate cancer has upregulation of a number of genes associated with cancer aggressiveness, progression free-survival, and metastasis [7]. When evaluating the clinical outcomes for these patients, overall survival for men with nonvisible Gleason 3 + 4 mirrored that for men with Gleason 3 + 3 disease, and it was only men with visible Gleason 3 + 4 cancer that fared worse [8]. Thus, one could hypothesise that the clinical significance of Gleason 3 + 4 cancer detected by systematic biopsies is not the same as the Gleason 3 + 4 cancer detected by MRI-TB. Furthermore, men with a negative MRI-TB can be safely monitored by a community doctor with interval prostate-specific antigen (PSA) testing, so that if a significant cancer was missed, these men are not lost to follow up.

What is an undisputed limitation of the addition of systematic biopsies to MRI-TB is the 34% higher odds of having insignificant cancer detected [6]. There is a major unmet need to reduce overtreatment brought about by a pathway based on PSA testing, and the detection of insignificant cancer is one of the primary drivers of this. While it is safe to monitor insignificant disease with active surveillance, this is expensive, costing just under US$30 000 per patient over 10 yr, and one-third of patients progress to radical treatment without progression in their disease status [9]. In addition, worldwide, approximately one in four men choose radical treatment in the absence of disease progression owing to the psychological and practical burden of protocol-based active surveillance.

Avoiding the addition of systematic biopsies leads to a quicker procedure time; a lower burden on the pathologist, who can thus spend more time on producing higher-quality analysis of fewer specimens; greater capacity in the urology service in a hospital; and, putatively, fewer side effects. When one considers that approximately 1 million biopsies are performed in Europe every year, these advantages accumulate.

There are a number of other considerations commonly cited for adding systematic biopsy in treatment decision planning such as the suitability of focal therapy and nerve-sparing decisions for radical prostatectomy. The mere presence of cancer on the contralateral side of the prostate, which is typically the information that proponents of systematic biopsy look for, does not preclude the patient from having nerve-sparing or focal therapy. It is more important to understand the location of that disease in relation to key anatomic structures such as the neurovascular bundle and urethral sphincter, which is information best gleaned from MRI. It has been demonstrated that the use of dedicated uroradiology MRI planning meetings before radical prostatectomy can improve patients’ functional outcomes and would not necessitate the addition of systematic biopsy [10].

In summary, systematic biopsies have a limited role in prostate cancer diagnosis. The cancer that is typically identified by systematic biopsies is not of the nature that is likely to benefit from treatment but can lead to expensive monitoring strategies and harmful patient side effects if overtreated.

  ***Conflicts of interest*:** Caroline M. Moore receives funding from Prostate Cancer UK, Movember, the Medical Research Council, Cancer Research UK, and the National Institute of Health Research (NIHR); receives fees for proctoring from SonaCare; has received speaker fees from Astellas and Janssen; and receives research support from Spectracure. Mark Emberton serves as a consultant/educator/trainer for Sonacare, Exact Imaging, Angiodynamics, and Profound Medical; and receives research support from the NIHR UCLH/UCL Biomedical Research Centre. Veeru Kasivisvanathan has nothing to disclose.

  ***Acknowledegments*:** Veeru Kasivisvanathan is an academic clinical lecturer and Caroline M. Moore is a research professor funded by the UK National Institute for Health Research (NIHR). Mark Emberton is an NIHR senior investigator (2015) and receives research support from the UCLH/UCL NIHR Biomedical Research Centre. The views expressed in this publication are those of the authors and not necessarily those of the NHS, the National Institute for Health Research, or the Department of Health.
